# Real-Time Optical Fiber Salinity Interrogator Based on Time-Domain Demodulation and TPMF Incorporated Sagnac Interferometer

**DOI:** 10.3390/s24165339

**Published:** 2024-08-18

**Authors:** Weihao Lin, Fang Zhao, Jie Hu, Yuhui Liu, Renan Xu, Xingwei Chen, Liyang Shao

**Affiliations:** 1 The Higher Educational Key Laboratory for Flexible Manufacturing Equipment Integration of Fujian Province, Xiamen Institute of Technology, Xiamen 361021, China; xurenan@xit.edu.cn; 2 School of Mechanical Electrical and Information Engineering, Xiamen Institute of Technology, Xiamen 361021, China; 3 Department of Electronic and Electrical Engineering, Southern University of Science and Technology, Shenzhen 518055, China; 12031197@mail.sustech.edu.cn (F.Z.); 12031313@mail.sustech.edu.cn (J.H.); 12068026@mail.sustech.edu.cn (Y.L.); 12232126@mail.sustech.edu.cn (X.C.); 4 The Peng Cheng Laboratory, Shenzhen 518005, China

**Keywords:** salinity monitoring, Sagnac interferometer, tapered polarization-maintaining fiber, optical time stretching technology, dispersion compensation fiber

## Abstract

A novel demodulation scheme for a point-type fiber sensor is designed for salinity concentration monitoring based on a Sagnac interferometer (SI) composed of a tapered polarization-maintaining fiber (TPMF) and optical time stretching technology. The SI, constructed using a PMF with a taper region of 5.92 μm and an overall length of 30 cm, demonstrated a notable enhancement in the evanescent field, which intensifies the interaction between the light field and external salinity. This enhancement allows for a direct assessment of salinity concentration changes by analyzing the variations in the SI reflection spectra and the experimental results indicate that the sensitivity of the sensor is 0.151 nm/‰. In contrast to traditional fiber optic sensors that depend on spectral demodulation with slower response rates, this work introduces a new approach where the spectral shift is translated to the time domain, utilizing a dispersion compensation fiber (DCF) with the demodulation rate reaching up to 50 MHz. The experimental outcomes reveal that the sensor exhibits a sensitivity of −0.15 ns/‰ in the time domain. The designed sensor is anticipated to play a pivotal role in remote, real-time monitoring of ocean salinity.

## 1. Introduction

In the past few decades, the fiber optic sensor has gained widespread adoption across various fields, such as for temperature [1], pressure [2], electric field [3], and magnetic field [4], attributed to its low cost, convenient networking, resistance to electromagnetic interference, and strong environmental adaptability [5,6,7,8]. The traditional interferometric fiber optic sensor is the most practical and technically comprehensive fiber sensor. It typically consists of four structures: the Mach–Zehnder interferometer (MZI) [9,10], the Michelson interferometer (MI) [11,12], the Fabry–Perot interferometer (FPI) [13,14], and the SI [15,16]. Among them, the SI boasts numerous distinct advantages in the realm of marine inspection and monitoring [17,18]. The SI, rooted in the Sagnac effect, utilizes the intricate interplay of light interference to precisely detect and quantify physical parameters like temperature and strain. The instrument has a light source whose beam is bifurcated by a beam splitter into two distinct paths. These two light beams traverse the same closed loop but in opposing directions, completing a full circuit before converging once more. Crucially, any rotational angular velocity within the plane of this loop introduces a disparity in the optical paths traversed by the beams, leading to a variation in their phase relationship [19]. This subtle phase shift, in turn, gives rise to a discernible movement of interference fringes at their point of reunion, serving as a reliable indicator of the underlying physical change [20,21]. The advantages of the SI in ocean monitoring are specifically embodied in its optical path structure, which minimizes its response to external vibrations, airflows, and other external factors, making it extraordinarily resilient to interference. In addition, the SI boasts broadband functionality, covering a wide frequency range from low to high frequencies, thereby satisfying diverse application scenarios. Lastly, the structure of the SI facilitates remote signal transmission, providing significant advantages for sensors in terms of remote monitoring and data acquisition [22]. Jin et al. devised a multi-wavelength fiber ring laser cavity incorporated with an SI and an MZI hybrid interferometer to achieve long-distance multi-point acoustic positioning function, exhibiting significant potential for application in challenging marine environments [23]. Zhang et al. utilized the forward Brillouin scattering generated by radial sound waves in a large-area optical fiber to form a Sagnac loop for dual parameter measurement of temperature and acoustic impedance [24]. Although fiber hydrophones are capable of assessing the state and fluctuations in the marine environment by capturing data like frequency, intensity, and variation patterns of oceanic sound waves, accurate reflection of the intensity and frequency of various oceanic activities requires a correlation between the sound wave levels and oceanic water quality factors, including the degree of acidification, temperature, and salinity.

Salinity is of paramount importance in marine environmental protection, where monitoring salinity enables prompt detection of salt pollution in water bodies, thereby facilitating the implementation of appropriate control measures. Moreover, variations in salinity can significantly impact the living conditions of marine aquatic organisms, underscoring the critical role of salinity monitoring in safeguarding aquatic biodiversity. Additionally, salinity monitoring in oceanographic research aids in comprehending the physical and chemical properties of seawater, thereby serving as a scientific foundation for the sustainable development of marine resources. For the marine aquaculture industry, salinity is a key factor influencing the growth of marine organisms. By closely monitoring salinity levels, breeders can refine breeding conditions, ultimately enhancing the survival and growth rates of marine organisms [25]. Meanwhile, real-time salinity monitoring is crucial for marine conservation and management. It is not only related to the maintenance of marine ecological balance but also a key data source for climate prediction and disaster warning. Through real-time monitoring, we can quickly respond to changes in salinity, protect biodiversity, and ensure the safety of economic activities. In addition, real-time salinity data also assist scientific research, promote advances in marine technology, and provide solid support for sustainable development. Therefore, strengthening real-time salinity monitoring is of great significance for protecting the ocean and addressing climate change. The necessity of real-time salinity monitoring lies in its crucial role in marine protection, ecological research, and economic activity safety. Firstly, changes in salinity directly affect the survival and ecological balance of marine organisms, and real-time monitoring can detect and respond to potential ecological crises in a timely manner. Secondly, salinity data are an important basis for climate prediction and disaster warning and are of great significance for reducing disaster losses. Finally, in economic activities such as fisheries and shipping, salinity monitoring helps to ensure production safety and improve resource utilization efficiency. Therefore, real-time salinity monitoring is a necessary means to maintain ocean health and promote sustainable development. The salinity fiber sensor excels in marine monitoring due to its exceptional accuracy, robust corrosion resistance, unparalleled sensitivity, and other inherent characteristics [26,27,28]. These features guarantee stable and reliable performance in challenging marine environments. Furthermore, its high sensitivity allows for precise measurement of even the slightest salinity variations, crucial for supporting marine environmental protection and water-quality-monitoring efforts [29,30]. Its compact design, lightweight build, and ease of installation and maintenance enhance the convenience and efficiency of long-term, continuous salinity monitoring in the ocean [31,32,33]. Consequently, the fiber optic salinity sensor stands as a formidable choice with vast potential for applications in ocean monitoring. Chen et al. introduced an innovative approach for temperature and salinity monitoring, employing an MZI crafted from polydimethylsiloxane (PDMS) and a tapered fiber. By harnessing the exceptional thermal optical coefficient of PDMS and the extensive evanescent field of the tapered fiber, this method attained a remarkable salinity sensitivity of 1.43 nm/% and a temperature sensitivity of −2.21 nm/°C [34]. However, this method necessitates the application of an additional polymer coating, posing challenges in precisely controlling the thickness and range of the coating layer. The repeatability of this method may require further examination and validation. Wei et al. attained the optical fiber surface plasmon resonance (SPR) effect through depositing a gold film onto a grounded conical optical fiber, successfully achieving salinity measurement with a remarkable sensitivity of 0.6898 nm/‰ [35]. However, the utilization of gold film poses challenges in terms of durability, hindering its application for long-term ocean monitoring. Currently, the predominant point-type fiber optic salinity monitoring solutions primarily focus on enhancing sensitivity [36,37,38,39], which is inherently constrained by the instrumentation, resulting in a relatively sluggish demodulation rate when utilizing traditional spectrometer-based experimental approaches. This poses a challenge for real-time monitoring in dynamic marine environments. Consequently, there is a pressing need for a novel demodulation method that can significantly expedite the demodulation rate for salinity monitoring. Optical fiber time-domain sensing, leveraging its advantage of rapid demodulation, has been proposed for realizing real-time monitoring [40]. This technology allows for a direct observation of the system’s output response, eliminating the need for complex mathematical transformations or inferences. This directness enables users to promptly acquire real-time status information of the system. Furthermore, time-domain sensing accurately depicts the system’s dynamic performance, encompassing stability, accuracy, and responsiveness. Through precise time-domain analysis, the accuracy and reliability of measurement results are ensured. Additionally, time-domain-sensing technology can monitor and swiftly respond to changes in measured parameters in real-time, enabling the timely detection and resolution of potential issues. This real-time capability is particularly crucial in scenarios requiring rapid response. Lastly, optical fiber time-domain-sensing technology enables continuous monitoring, facilitating the acquisition of data variations and trends over extended periods, thereby enhancing the accuracy and reliability of monitoring.

In this work, we present a fiber sensor utilizing a time-stretching demodulation technique and the SI composed of TPMF, specifically tailored for measuring salinity concentration variations. This approach involves the use of dispersion-compensating fiber (DCF) to translate the interference spectrum movements caused by alterations in salinity concentration from the spectral domain to the time domain with an impressive interrogation speed of up to 50 MHz. The TPMF with a waist width of 5.92 μm acts as a sensitization fiber, augmenting the interaction between evanescent waves and external salinity which allows for highly sensitive measurements. Experimental results reveal that the sensor achieves a salinity sensitivity of 0.151 nm/‰ in the spectral domain and 0.15 ns/‰ in the time domain. The designed sensing system stands out for its swift interrogation speed, remarkable sensitivity, and cost-effectiveness, positioning it as a potential new standard in ocean salinity monitoring for real-time seawater concentration tracking.

## 2. Working Principle and Experimental Setup

**Figure 1:**
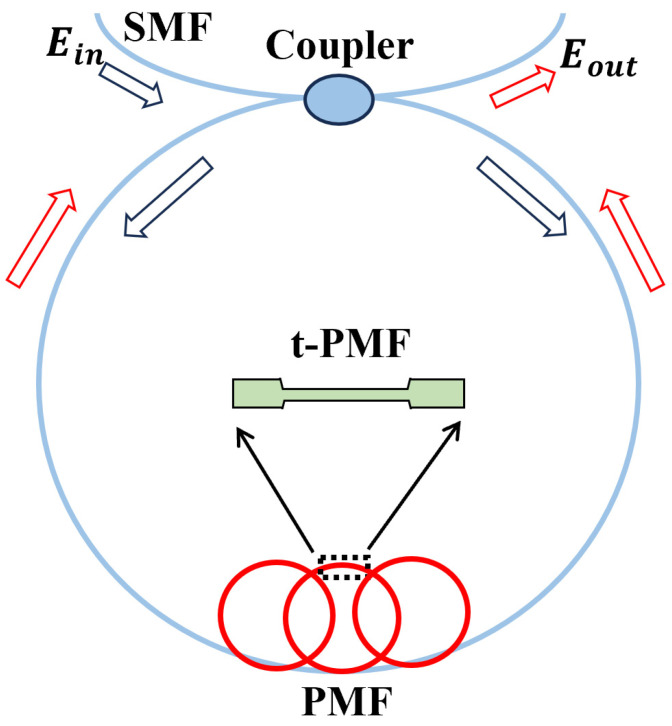
The schematic diagram of the Sagnac interferometer (SI) structure composed of tapered polarization-maintaining fiber (TPMF). Red arrows: light output; Blue arrows: light input.

Figure 1 shows the schematic diagram of an SI composed of a tapered PMF. The laser source passes through a 3 dB coupler and is then evenly divided into two beams. The beams propagate simultaneously in the loop, one clockwise and the other counterclockwise. Upon their return to the 3 dB coupler, the beams interfere with each other due to the difference in optical path. Additionally, the high birefringence B of PMF introduces a notable phase shift during transmission, which can be mathematically expressed as [41]:

(1) $$\varphi_{PMF}=2\pi \frac{BL}{\lambda }$$

Here, *L* denotes the length of the PMF, while *λ* is the center wavelength of the laser which is set at 1560 nm.

**Figure 2:**
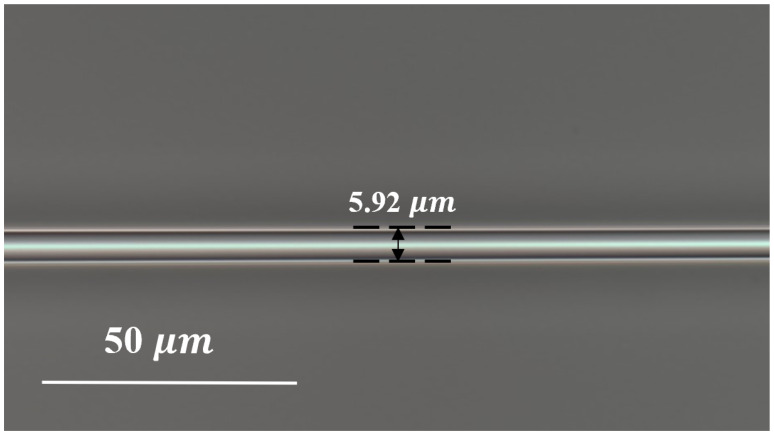
The microscopic structure diagram of TPMF.

Utilizing tapered fiber technology, the evanescent field of the fiber core is brought into full contact with the external refractive index (RI), enabling accurate salinity measurement, and the microstructure of TPMF is shown in Figure 2, with a width of 5.92 μm. At the tapered region, the input light intensity splits, with a fraction coupling to the surrounding environment and the remainder remaining within the fiber. Upon convergence of these two light fractions in the conical region, a phase difference arises due to the discrepancy between the RI of the environment and the RI of the fast and slow axes of the PMF, denoted as $\Delta n_{eff}$. This phase difference can be mathematically described as [41]:

(2) $$\varphi_{tPMF}=2\frac{\pi l}{\lambda }\Delta n_{eff}$$

where *l* represents the effective length of the RI-sensitive structure. The phase shifts trigger optical interference, and the output is captured by both an optical spectrum analyzer (OSA) and a photo detector (PD).

The transmittance of the system can be written as [39]:

(3) $$T=\left[{\left|E_{Tx}\right|}^{2}+{\left|E_{Ty}\right|}^{2}\right]/\left[{\left|E_{x}\right|}^{2}+{\left|E_{y}\right|}^{2}\right]$$

In this equation, $E_{x}$, $E_{y}$, $E_{Tx}$, and $E_{Ty}$ refer to the initial and transmitted electric field components under an orthogonal polarization mode. To articulate the result of the electric field transmission, a Jones matrix is employed under the assumption of negligible loss, which is defined by [41]:

(4) ETxETy=θ1θ2Jcw−θ2′θ1′Jccwθ1′ExEy

where $\theta_{1}$, $\theta_{2}$, $\theta_{1^{\prime }}$, and $\theta_{2^{\prime }}$ represent the polarization rotation angles along the polarization axis at the two splicing points between the PMF and the single-mode fiber (SMF) in the SI, considering both clockwise and counterclockwise directions. $J_{cw}$ and $J_{ccw}$ denote the Jones matrices for light propagating through the TPMF in the clockwise and counterclockwise directions, respectively, which can be written as [41]:

(5) $$J_{cw}\approx J_{ccw}=\left(\begin{array}{cc} e^{-\frac{j\varphi_{tPMF}}{2}} & 0\\ 0 & e^{\frac{j\varphi_{tPMF}}{2}} \end{array}\right)\left(\begin{array}{cc} e^{-\frac{j\varphi_{PMF}}{2}} & 0\\ 0 & e^{\frac{j\varphi_{PMF}}{2}} \end{array}\right)$$

Based on the aforementioned equations, it has been established that the output transmission is influenced by the RI difference between the fiber sensor and its surroundings, indicating that salinity variations can be tracked accordingly. Specifically, the RI sensitivity can be expressed as:

(6) $$S_{RI-tPMF}=\frac{\Delta \lambda }{\Delta n}=\frac{d\Delta n_{eff}\cdot \lambda }{dn\cdot \Delta n_{eff}}$$

**Figure 3:**
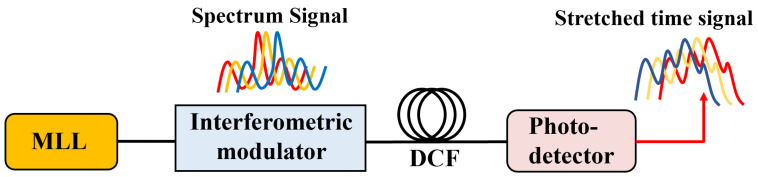
The schematic diagram of time-domain sensing based on dispersion compensation fiber (MLL: mode-locked laser).

It is well-established that a linear mapping of wavelength to time can be attained through the utilization of DCF exhibiting linear dispersion, as illustrated in Figure 3. The correlation between wavelength shifts influenced by external RI and their corresponding time domain shifts is expressed by the following equation [40]:

(7) Δt=D·Δλ

In which *D* is the dispersion coefficient of DCF equal −1000 ps/nm and Δ*t* is the offset of the time-domain spectrum. The relationship between the wavelength and its corresponding time is evident from Equation (7). Due to the negative dispersion coefficient of DCF, the changes in the time domain are negatively correlated with the shifts in the interference peaks in the wavelength domain.

**Figure 4:**
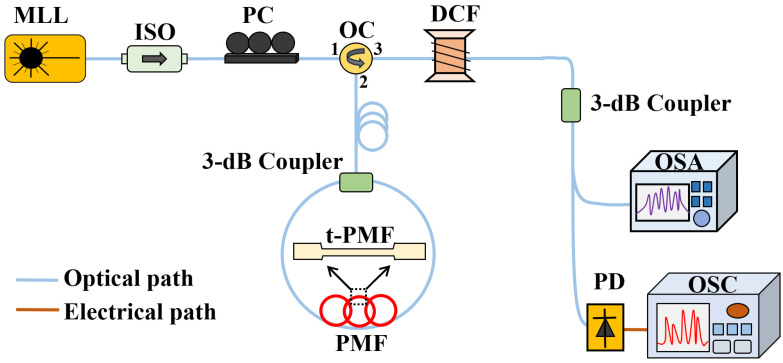
The experimental setup of the designed ultra-fast salinity fiber sensor.

The experimental setup is illustrated in Figure 4. A mode-locked fiber laser (MLL) (EFLA0132, ROI, Guangdong, China) emits femtosecond pulses (44 fs) with a wavelength of 1560 nm and a repetition rate of 50 MHz, which are transmitted to the optical coupler (OC) through an isolator (ISO) and polarization controller (PC). The ISO is employed to safeguard the light source from the harm caused by backscattered light, whereas the PC is utilized to regulate the polarization state of light to guarantee the output light intensity. The pulse light is transmitted via the OC to the 3 dB coupler, where it is simultaneously directed both forward and backward towards the TPMF and it interacts with varying salinity concentrations. Upon recoupling at the coupler, this interference is then transmitted through the OC to the DCF, successfully mapping the spectral domain interference to the time domain. The pulse signal carrying interference information is broadened by DCF and transmitted to the OSA (AQ6370D, YOKOGAWA, Japan) and oscilloscope through a 3 dB coupler for both wavelength- and time-domain demodulation.

## 3. Experimental Results

**Figure 5:**
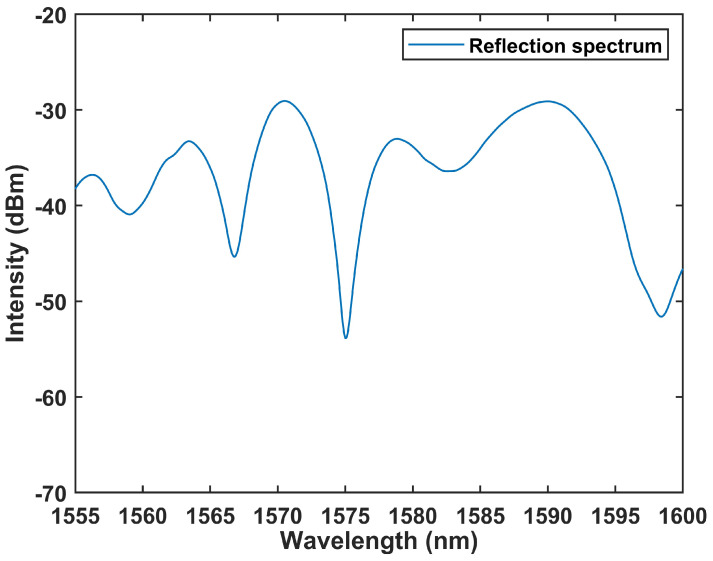
The reflection spectrum at output port based on spectral demodulation.

Figure 5 depicts the reflection spectrum of the SI that comprises the TPMF, fabricated using a tapered machine (AFBT-8000LE-H0, Kepler, Shandong, China). The TPMF, heated via hydrogen ignition, has a total length of 25 mm and a waist width of 5.92 μm. The total length of PMF is 30 cm. The specific process for fabricating a tapered optical fiber is as follows: first, the protective coating of the PMF is stripped off, it is cleaned with alcohol, and then it is placed on the tapering machine. Secondly, the vacuum pump is turned on to create a vacuum and use magnets to thoroughly adsorb the optical fiber onto the machine. Thirdly, the operation interface is initialized, the hydrogen ignition device is turned on, and the heating nozzle is ignited to preheat the optical fiber. Fourthly, the upper computer is controlled to start stretching the optical fiber, and the stretching length is fixed at 25 mm to obtain the required tapered optical fiber. Additionally, Figure 5 highlights the superior interference effect, achieving a signal-to-noise ratio (SNR) exceeding 20 dB across the spectrum.

**Figure 6:**
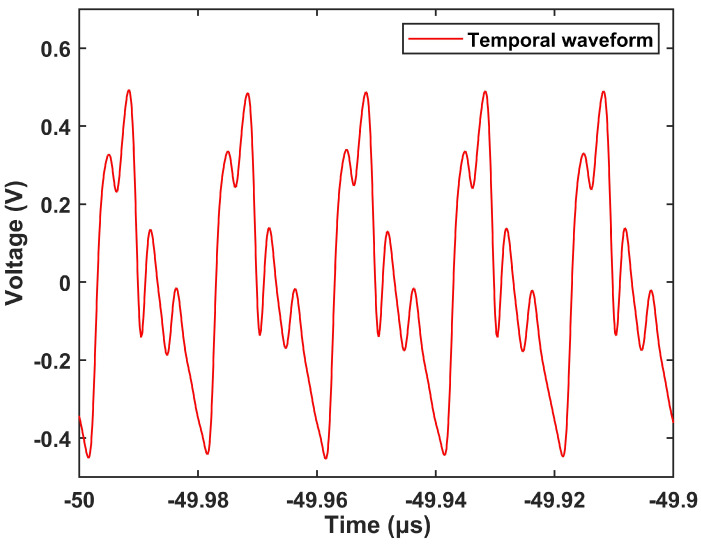
The temporal waveform based on time domain demodulation.

Figure 6 exhibits the time waveform resulting from the mapping of wavelength data from the spectral domain to the time domain. The results indicate notable interference effects in the time domain with some interference information absent. The situation likely stems from the limited dispersion coefficient of the DCF, which fails to fully capture the spectral information. To address this, substitution of the DCF with a higher-dispersion-coefficient fiber is able to solve the problem. Despite this limitation, the interference spectrum in the time domain remains sensitive to salinity variations, as evidenced by subsequent experimental findings.

**Figure 7:**
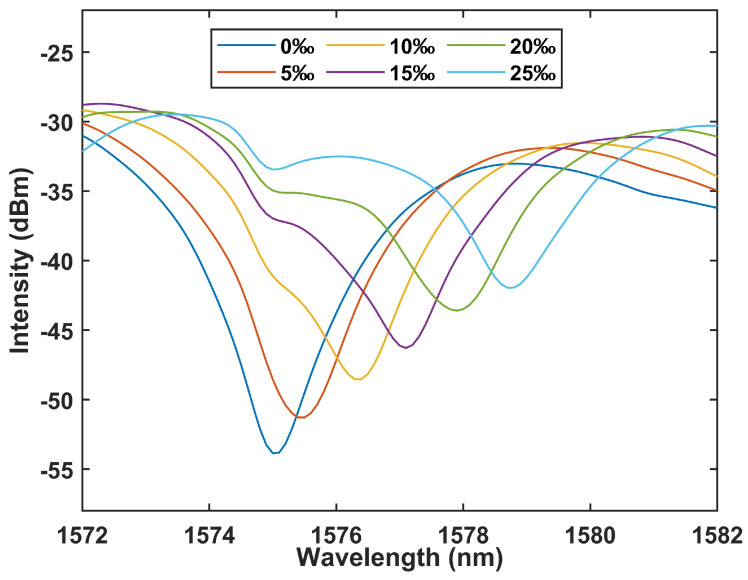
The reflection spectra of the fiber sensor under different concentrations of salinity.

Figure 7 depicts the variation in the interference spectrum shift as the salinity concentration escalates from 0‰ to 25‰, exhibiting a redshift towards longer wavelengths at a 5‰ incremental increase in concentration. This alignment concurs with the theoretical underpinnings outlined in Equation (7).

**Figure 8:**
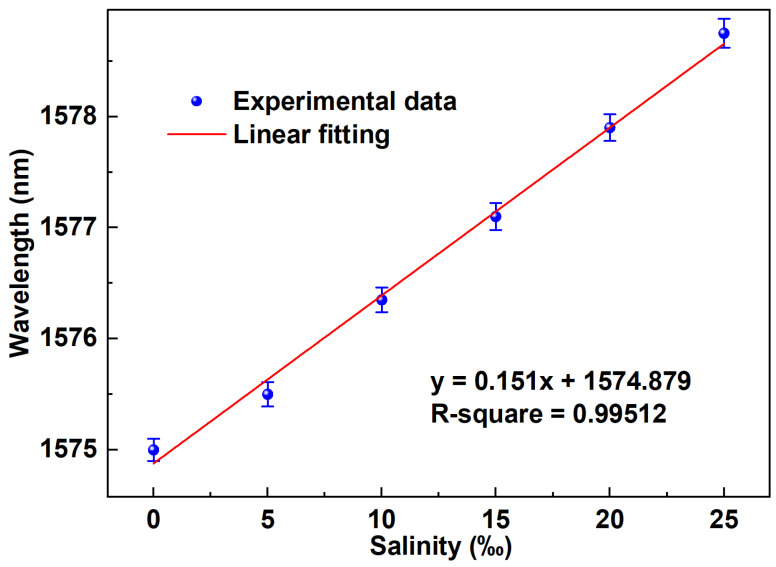
The linear fitting curve depicts the reflection spectrum of the salinity sensor.

Figure 8 presents a schematic visualization of the linear fitting equation in the spectral domain. The designed salinity sensor exhibits superior sensitivity, achieving a sensitivity of 0.151 nm/‰. The coefficient of determination, reaching 0.99512, demonstrates the sensor’s outstanding linear response and robust ability to demodulate wavelength shifts resulting from salinity changes within the range of 0‰ to 25‰. This significant accomplishment lays the foundation for ultra-fast demodulation techniques based on the time domain.

**Figure 9:**
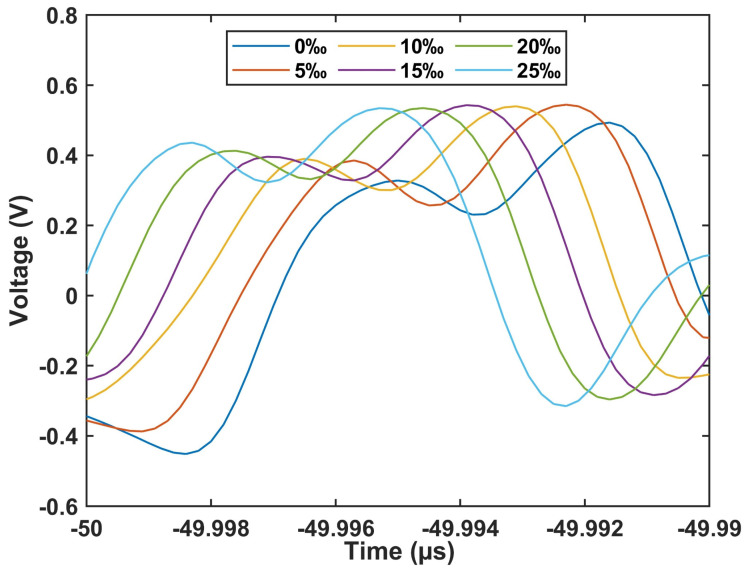
The temporal waveform of the fiber sensor under different concentrations of salinity.

Figure 9 illustrates the interference waveform in the time domain, revealing that as salinity concentration rises, the waveform undergoes a blue shift towards shorter wavelengths, contrary to the trend observed in Figure 7. This phenomenon arises due to the negative dispersion coefficient of DCF, aligning with the theoretical predictions outlined in Equation (7).

**Figure 10:**
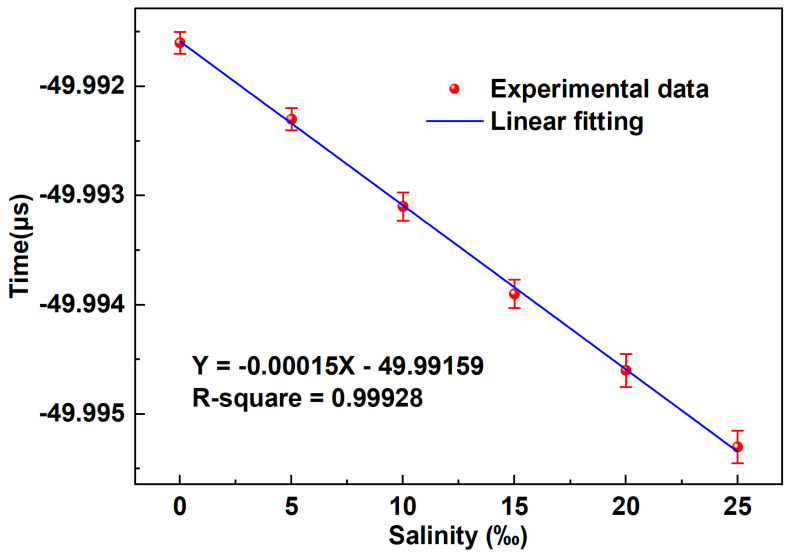
The linear fitting curve depicts the temporal waveform of the salinity sensor.

**Figure 11:**
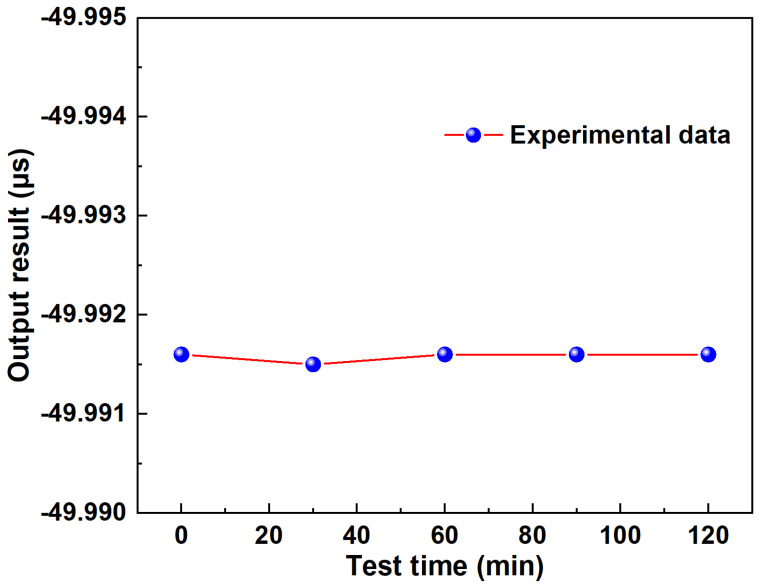
The Stability testing of sensor in the time domain (at a concentration of 0‰).

Figure 10 presents the outcome of the linear fitting equation applied to the time waveform, exhibiting a noteworthy sensitivity of −0.15 ns/‰ in the time domain, indicating its superior performance. The coefficient of determination stands at an impressive 0.99928, reflecting an almost perfect linear relationship. This remarkable linearity is highly beneficial for the sensor, facilitating intuitive assessments of salinity concentration. Figure 11 shows the stability test results of the sensor, and the experimental results show that when the salinity concentration is 0‰, the fluctuation in the time domain is less than one thousandth of a microsecond. Furthermore, leveraging the ultra-high repetition rate of the femtosecond pulse, this system achieves an interrogation demodulation speed of 50 MHz, surpassing an ordinary OSA by hundreds of times in speed. The experiment is repeated five times and the maximum error in time domain is calculated to be 0.00015, and the maximum error in wavelength domain is calculated to be 0.13.

## 4. Discussion

The upper limit of the demodulation rate of traditional point-type optical fiber sensors is 20 KHz, while the salinity sensor we designed has achieved ultra-fast demodulation with a rate up to 50 MHz based on the time-stretch method which is a thousand-fold increase. In addition, compared to our previous work [41], the SI-based salinity sensor enables ultra-long-distance monitoring through the cascade of couplers and circulators, whereas the MZI structure has significant limitations in sensing distance, restricting its application scenarios primarily to laboratory environments. Consequently, the SI structure is more practical for real-time ocean salinity monitoring. Nonetheless, in the realm of fiber optic ocean salinity monitoring, three principal challenges persist: firstly, the tapered region of the fiber is particularly vulnerable, prone to damage in the intricate marine environment. Secondly, the oscilloscope’s data collection capacity is limited, often resulting in data saturation during prolonged data gathering. Lastly, fiber optic interferometers exhibit cross-sensitivity, posing a risk of significant measurement inaccuracies. The aforementioned challenges are not intractable. To address the fragility of fiber, the sensor can be enhanced by encapsulating it with polymers and metal materials [42,43]. To overcome the limitation of data access, data acquisition cards can be employed for remote, real-time data collection [44,45]. Furthermore, neural network algorithms can be utilized to train and analyze wavelength shifts and intensity changes induced by multiple parameters, thereby mitigating cross-sensitivity issues [46,47]. Meanwhile, the designed sensor inevitably exhibits cross-sensitivity, a phenomenon also observed in point-type optical fiber sensors. In practical applications, temperature alters the length and refractive index of optical fibers through thermal expansion and contraction, contaminants change the refractive index and absorption characteristics of the fibers, and water flow may modify the fixed state of the fibers, optical path length, and light scattering, all of which lead to changes in light wave phase and subsequently affect the stability and accuracy of interference signals. To tackle these issues, measures such as temperature compensation technology, temperature control, anti-fouling coatings, encapsulation protection, regular cleaning, fixed design, flow field control, and protective shields can be employed. These measures effectively mitigate the impacts of temperature, contaminants, and water flow on the fiber optic interferometric salinity sensor, thereby enhancing its stability and accuracy.

The selection of demodulation techniques for fiber optic interferometric sensors involves a trade-off between spectral demodulation and time-stretch demodulation in the time domain. Spectral demodulation stands out due to its high resolution and ability to capture comprehensive spectral information, making it crucial for applications requiring precise spectral measurements. However, this technology often comes with high costs, relatively slow scanning speeds, and large equipment sizes, limiting its applicability in cost-sensitive or highly portable scenarios. In contrast, time-stretch demodulation in the time domain has garnered significant attention for its low cost, high-speed response, and ease of integration. It is particularly suitable for applications demanding real-time performance, miniaturization, and portability. Nevertheless, it is important to note that despite its numerous advantages, time-stretch demodulation may have limitations in demodulation accuracy and technical complexity, which may render it unsuitable for all types of fiber optic interferometric sensors, thus limiting its application range.

Table 1 compares the sensitivity of our sensor with that of previously reported sensors. As can be seen from the table, our sensor has a sensitivity that is comparable to other optical fiber sensors, while also being able to achieve ultra-fast salinity monitoring.

## 5. Conclusions

In conclusion, a novel real-time fiber optic salinity sensor based on the time-stretching effect and the SI composed of TPMF has been theoretically demonstrated and experimentally validated. The laser pulse is simultaneously transmitted in both clockwise and counterclockwise directions through the coupler to the TPMF, which possesses a waist width of 5.92 μm. This configuration generates interference effects and effectively amplifies the reaction between the evanescent field and external salinity concentration, ultimately resulting in a salinity sensitivity of 0.151 nm/‰. DCF seamlessly transitions the interference from the spectral domain to the time domain, achieving a sensitivity of −0.15 ns/‰. Furthermore, leveraging the high repetition rate of femtosecond lasers, the demodulation rate of the system soars to 50 MHz, outperforming traditional spectral demodulation methods by hundreds of times. The designed sensor boasts advantages including a rapid demodulation rate, superior sensitivity, a streamlined structure, and cost-effectiveness, making it a promising candidate for marine environmental monitoring.

## Figures and Tables

**Table 1:** The sensitivity comparation with different types of salinity sensor.

Structures	Salinity Sensitivity (nm/‰)	Ref.
MZI with PDMS	0.143	[32]
Balloon-shaped SMF	0.168	[48]
Asymmetric MZI	0.309	[49]
LPFG	0.125	[50]
This work	0.151	/

## Data Availability

Obtained experimental data are available from the corresponding author upon reasonable request.
